# Primary Infectious Mononucleosis Masquerading as Post-operative Fever in a Young Patient with Cemento-ossifying Fibroma of the Skull Base

**DOI:** 10.7759/cureus.6327

**Published:** 2019-12-08

**Authors:** Ryan M Edelbrock, Bicky Thapa, Michael A Fritz, Pablo Recinos, Hamid Borghei-Razavi

**Affiliations:** 1 Neurosurgery, Cleveland Clinic, Cleveland, USA; 2 Hematology/Oncology, Medical College of Wisconsin, Milwaukee, USA; 3 Otolaryngology, Head and Neck Institute, Cleveland Clinic, Cleveland, USA; 4 Neurosurgery, Taussig Cancer Center, Cleveland Clinic, Cleveland, USA; 5 Neurosurgery, Neurological Institute, Cleveland Clinic - Taussig Cancer Center, Cleveland, USA

**Keywords:** epstein-barr virus infectious mononucleosis, meningitis, skull base surgery, post-operative, post-operative fever

## Abstract

The typical presentation of infectious mononucleosis (IM) is characterized by a triad of fever, pharyngitis, and lymphadenopathy. Epstein-Barr virus (EBV) is the most common etiologic agent for IM. Humans are the reservoir for EBV, and it is transmitted via intimate contact between individuals.

This case presents a 19-year-old male with recurrent cemento-ossifying fibroma of the skull base with a complicated post-operative course including bacterial meningitis, cerebrospinal fluid (CSF) leak, and intermittent fevers despite treatment with intravenous cefepime. Head computed tomography (CT) revealed a nonspecific subdural collection that could represent an empyema. However, exploratory craniotomy revealed no empyema. CT chest demonstrated bilateral hilar mediastinal lymphadenopathy and splenomegaly. Blood work for fever of unknown origin was positive for EBV immunoglobulin M, and EBV deoxyribonucleic acid 180,565 IU/mL.

The diagnosis of EBV IM in this case was elusive because it presented post-operatively, symptoms aligned with the patient’s CSF leak, and he reported no sexual or sick contacts. For post-operative young patients with recurrent fevers of unknown origin, it is important to consider EBV IM in the differential. Earlier diagnosis could have saved the patient unneeded tests, prevented surgical re-exploration, and resulted in a shorter hospital stay.

## Introduction

The typical presentation of infectious mononucleosis (IM) is characterized by a triad of fever, pharyngitis, and lymphadenopathy. Other clinical manifestations include malaise, fatigue, nausea, anorexia, myalgias, arthralgia, headache, splenomegaly, bradycardia, hepatomegaly, jaundice, skin rash, and pneumonitis [1]. Epstein-Barr virus (EBV), also known as human herpesvirus 4, is the most common etiologic agent for IM. Humans are the reservoir for EBV, and it is transmitted via intimate contact between individuals. The incubation period for IM is approximately four to seven weeks. Although EBV IM can affect any age, it is most common in young adults [2].

One of the epidemiological studies showed the overall EBV seroprevalence of 66.5% in children aged between 6 and 19 years in the United States. The authors also reported higher seroprevalence of EBV with increasing age; 54.1% in 6-9 years vs 82.9% for 18-19 years [2]. Globally, EBV is highly prevalent, and it is estimated to be positive in more than 90% of the world’s population [3]. 

EBV is a lymphotropic herpes virus that initially infects epithelial cells followed by naïve B-cell infection, where it persists for life in a latent state [3]. EBV strains are classified as type A and B (or type 1 and 2) based on EBV nuclear antigen-2 (EBVNA2) gene sequence [4]. EBVNA2 gene plays a vital role in the activation and proliferation of host B cell.

We report a challenging case of primary IM presenting as unremitting fever in the setting of cerebrospinal fluid (CSF) rhinorrhea in a young patient who had undergone multiple skull base operations. The purpose of this case is to identify the atypical presentation of the EBV IM in complicated post-operative patients.

The patient has provided permission to publish the features of his case, and the identity of the patient has been protected.

## Case presentation

A 19-year-old male with recurrent cemento-ossifying fibroma of the skull base, a rare skull base tumor, underwent right orbitofrontal skull base craniotomy for resection of the ossifying fibroma and reconstruction of orbital rim and roof with bone grafts. Post-operative course was complicated by CSF rhinorrhea one week post-operatively, which was managed with the placement of a lumbar subarachnoid drain. However, his clinical course further deteriorated with the development of headaches, high-grade fever, neck pain, and altered mental status. Leukocytosis was present on blood workup, computed tomography (CT) scan of the head showed no acute process, CSF analysis (Table 1) showed white blood cell (WBC): 6,389, neutrophil: 80%, protein: 249, glucose: 30; culture was positive for Enterobacter cloacae. The patient was managed for bacterial meningitis with intravenous (IV) cefepime; the lumbar drain was removed and he was discharged on IV cefepime. However, he continued to have intermittent positional headaches and postnasal drip with a metallic taste. The nasal discharge was positive for beta 2 transferrin indicating CSF leak, which was urgently repaired with endoscopic surgery one month after surgery. He was also noted to be intermittently febrile despite being on IV cefepime for bacterial meningitis.

**Table 1:** CSF Analysis CSF, cerebrospinal fluid; RBC, red blood cell; H, high; L, low; µL, microliter; mg/dL, milligrams per deciliter

CSF	Reference Range	
Color	Colorless	Neon
Supernatant color	Colorless	Neon
Clarity	Clear	Slightly turbid
Supernatant clarity	Clear	Clear
RBC	0-1 /µL	13 (H)
Total nucleated cells	0-5 /µL	6,389 (H)
Neutrophil %	0%-3%	80 (H)
Lymphocyte%	50%-90%	3 (L)
Monocyte%	10%-50%	17
Protein	15-45 mg/dL	249 (H)
Glucose	40-70 mg/dL	30 (L)

After successful endoscopic repair, the patient remained intermittently febrile. On examination, the patient stated he had not had any sick contacts, nor was he sexually active. CT head demonstrated a small nonspecific subdural collection that could possibly represent empyema in the area of the reconstruction as well as an encephalocele in the skull base reconstruction site. However, an exploratory craniotomy failed to reveal any empyema. The total WBC was 20,000 (mostly lymphocytes). CT scan of the chest showed bilateral hilar mediastinal lymphadenopathy, and splenomegaly.

 

**Figure 1:**
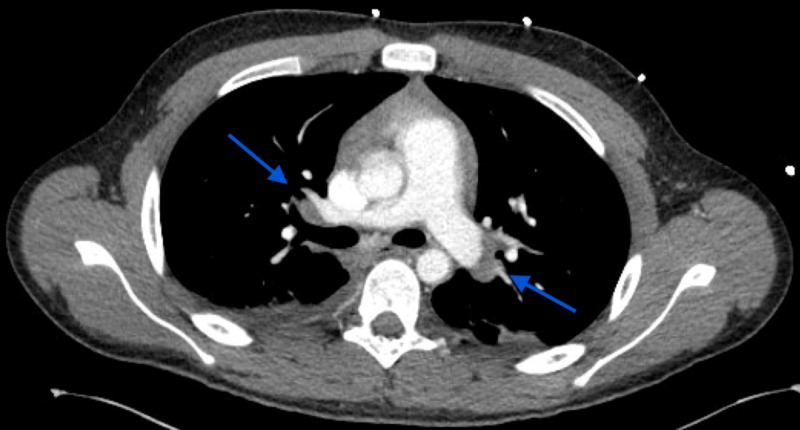
CT Chest Arrows indicate bilateral hilar lymphadenopathy

**Figure 2:**
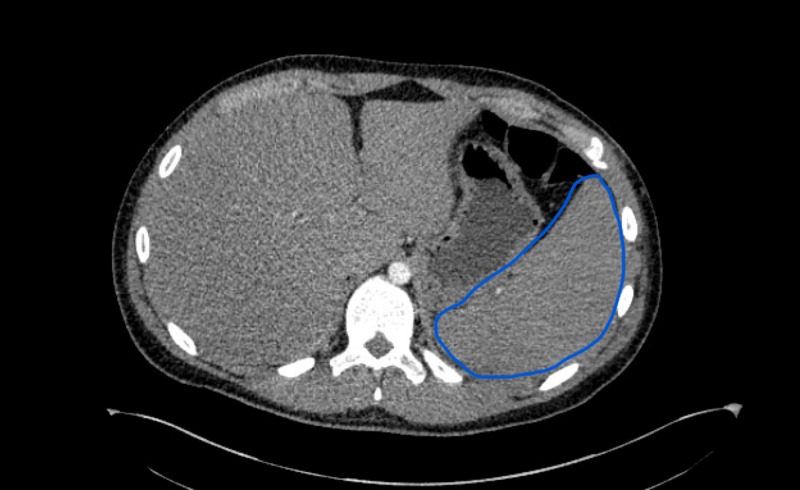
CT Chest Blue outline indicates splenomegaly

Further blood workup for fever of unknown origin was positive for EBV immunoglobulin M (IgM), and EBV deoxyribonucleic acid was 180,565 IU/mL (Table 2). It was concluded that our patient had primary IM masquerading as post-operative fever. EBV IM developed between Enterobacter meningitis and the endoscopic CSF leak repair. The patient was managed conservatively, fever improved, and he was discharged.

**Table 2:** EBV antibody panel and polymerase chain reaction EBV, Epstein-Barr virus; VCA, viral capsid antigen; EA, early antigen; IgM, immunoglobulin M; IgG, immunoglobulin G; Qual, qualifier value

EBV VCA, IgG	0.2
EBV VCA, IgM	>4.0
EBV nuclear antigen antibody	<0.2
EBV EA antibody	0.4
EBV VCA IgG, Qual	Negative
EBV VCA IgM, Qual	Positive

## Discussion

The diagnosis of IM was elusive in this case because it presented post-operatively and the symptoms aligned with the patient’s CSF leak. Although the patient was in the age range for the development of primary IM, he did not have the classic presentation of IM [1]. The only clues for primary IM were the patient’s recurrent fever and splenomegaly.

EBV is primarily transmitted through saliva; however, blood transfusion and organ transplantation are also risk factors for EBV transmission [5,6]. Additionally, EBV is associated with various malignancies such as nasopharyngeal carcinoma, Burkitt’s lymphoma, post-transplant lymphoproliferative disease, gastric carcinoma, and Hodgkin’s lymphoma [7-10].

Patient suspicious of EBV IM should be evaluated with complete blood count with differentials. Lymphocytosis is a common finding in patients with IM and is associated with circulating elevated atypical lymphocytes. Reactive heterophile antibodies in patients with IM are diagnostic, and no further testing is required. 

Typically, EBV infection in childhood is asymptomatic or manifest with mild symptoms as other common illnesses. After primary infection, EBV goes into the latency phase. EBV IM in young patients usually presents with one to two weeks of symptoms followed by uneventful recovery. Clinical manifestations of EBV IM in adult patients include fever, malaise, fatigue, sore throat, nausea, cough, headache, myalgia, arthralgia, rash, lymphadenopathy, splenomegaly, and hepatomegaly.

Complicated life-threatening EBV IM includes encephalitis, splenic rupture, acute liver failure, myocarditis, renal dysfunction, hemophagocytosis, and airway obstruction [11]. Monitoring the patient’s cognitive state is essential to identify encephalitis. It is also essential to monitor the patient for hypertriglyceridemia, elevated soluble CD25, low ferritin, and peripheral blood cytopenia. These findings associated with the patient’s high fever, lymphocytosis, and splenomegaly would suggest adult hemophagocytosis, a complication of EBV [12]. While encephalitis and hemophagocytosis are rare complications of EBV, it was important to consider in this patient since he was immunosuppressed given his post-operative, post-infectious state [13].

## Conclusions

For post-operative young patients with recurrent fevers of unknown origin, it is crucial to consider IM in the differential diagnosis. Although the patient’s presentation was not classic for primary IM, the earlier diagnosis might have saved the patient’s unneeded tests, prevented surgical re-exploration, and resulted in a shorter hospital stay, post CSF leak repair. Diagnosing IM in post-operative patients with a CSF leak can be elusive due to the overlap of signs and symptoms. Still, considering IM in the differential for young post-operative patients can lead to improved outcomes and reduced costs.
